# TAK1 (MAP3K7) Signaling Regulates Hematopoietic Stem Cells through TNF-Dependent and -Independent Mechanisms

**DOI:** 10.1371/journal.pone.0051073

**Published:** 2012-11-30

**Authors:** Giichi Takaesu, Maiko Inagaki, Keiyo Takubo, Yuji Mishina, Paul R. Hess, Gregg A. Dean, Akihiko Yoshimura, Kunihiro Matsumoto, Toshio Suda, Jun Ninomiya-Tsuji

**Affiliations:** 1 Center for Integrated Medical Research, Keio University School of Medicine, Tokyo, Japan; 2 Department of Environmental and Molecular Toxicology, North Carolina State University, Raleigh, North Carolina, United States of America; 3 Department of Cell Differentiation, The Sakaguchi Laboratory of Developmental Biology, Keio University School of Medicine, Tokyo, Japan; 4 Department of Biologic and Materials Sciences, School of Dentistry, University of Michigan, Ann Arbor, Michigan, United States of America; 5 Department of Clinical Sciences, College of Veterinary Medicine, North Carolina State University, Raleigh, North Carolina, United States of America; 6 Center for Comparative Medicine and Translational Research, College of Veterinary Medicine, North Carolina State University, Raleigh, North Carolina, United States of America; 7 Department of Microbiology and Immunology, Keio University School of Medicine, Tokyo, Japan; 8 Department of Molecular Biology, Graduate School of Science, Nagoya University, Nagoya, Japan; University of Frankfurt - University Hospital Frankfurt, Germany

## Abstract

A cytokine/stress signaling kinase *Tak1* (*Map3k7*) deficiency is known to impair hematopoietic progenitor cells. However, the role of TAK1 signaling in the stem cell function of the hematopoietic system is not yet well defined. Here we characterized hematopoietic stem cells (HSCs) harboring deletion of *Tak1* and its activators, *Tak1* binding proteins 1 and 2 (*Tab1* and *Tab2*) using a competitive transplantation assay in a mouse model. *Tak1* single or *Tab1*/*Tab2* double deletions completely eliminated the reconstitution activity of HSCs, whereas *Tab1* or *Tab2* single deletion did not cause any abnormality. *Tak1* single or *Tab1*/*Tab2* double deficient lineage-negative, Sca-1^+^, c-Kit^+^ (LSK) cells did not proliferate and underwent cell death. We found that *Tnfr1* deficiency restored the reconstitution activity of *Tak1* deficient bone marrow cells for 6–18 weeks. However, the reconstitution activity of *Tak1*- and *Tnfr1*-double deficient bone marrow cells declined over the long term, and the number of phenotypically identified long-term hematopoietic stem cells were diminished. Our results indicate that TAB1- or TAB2-dependent activation of TAK1 is required for maintenance of the hematopoietic system through two mechanisms: one is prevention of TNF-dependent cell death and the other is TNF-independent maintenance of long-term HSC.

## Introduction

Hematopoiesis is maintained by self-renewal of hematopoietic stem cells (HSCs) and differentiation and proliferation of HSC-derived hematopoietic progenitors [1]–[3]. Actively proliferating bone marrow cell populations include short-term repopulating HSCs (ST-HSCs) and multipotent progenitors, MPPs. Phenotypically both populations are lineage negative, Sca1^+^ c-Kit^+,^ (LSK), and CD34^+^, while ST-HSCs are Flt3^−^ and MPPs are Flt3^+^. Although ST-HSCs and MPPs can provide multilineage hematopoietic cells for a short term [4], hematopoietic pluripotency is maintained by the less frequently self-renewed population of HSCs: long-term multilineage reconstituting hematopoietic stem cells (LT-HSCs) that are phenotypically comprised of CD34^−^, LSK or CD150^+^, LSK [1], [5]. Several recent studies have revealed that the protection from stress-mediated cell damage is critical for HSC maintenance [6]. For example, the FOXO family of transcriptional factors, which regulate stress responses, are essential for HSC maintenance [7]. Deficiency of DNA damage sensor, Atm1, abolishes HSC function [8]. Recently, chromatin remodeling factor, Bmi1, was found to be indispensable for HSC function by modulating DNA damage signaling and oxidative stress [9]. A protein kinase LKB is reported to play a unique role in maintenance of HSCs by preventing oxidative stress-induced mitochondrial dysfunction [10]–[12]. While the importance of stress responses in HSCs is clear, the signaling pathways to protect HSCs from cell damage are still largely unknown.

TAK1 (MAP3K7) is a member of mitogen-activated protein kinase kinase kinases (MAPKKK), and activated by inflammatory cytokines and stress conditions. TAK1 and TAK1 binding proteins 1 and 2 (TAB1 and 2) are major components of the TAK1 complex, which can activate MAPK cascades as well as the transcription factor NF-κB. TAB1 and TAB2 are structurally unrelated and bind to TAK1 at different sites [13], [14]. TAB2 and its closely related protein, TAB3, confer ubiquitin binding domains and tethers between TAK1 and the polyubiquitin chain, resulting in activation of TAK1 in cytokine signaling pathways [15]–[19]. TAB2 and TAB3 can redundantly function in cytokine signaling pathways, but TAB2 is indispensable during development [20]. We recently demonstrated that TAB1 is involved in stress-dependent TAK1 activation [21] and the basal activity of TAK1 in epithelial tissues [22]. TAK1 is critical in modulating reactive oxygen species and preventing cell death in epithelial cells *in vivo* [23]–[26]. Thus, the stress- and cytokine-activated TAK1 complex is one of the pathways to protect cells from reactive oxygen species at least in the epithelium. Based on this, we hypothesized that the TAK1 complex participates in protection of HSC and may be involved in HSC maintenance. Inducible *Tak1* deficiency in hematopoietic cells and hepatocytes has recently been reported to cause cell death within 12 days after induction of gene deletion through a TNF-dependent mechanism [27], [28]. This short term assay demonstrates that TAK1 is important for preventing cell death in ST-HSCs and MPPs. However, the involvement of TAK1 signaling in stem cell function, specifically the ability of long-term reconstitution, has not yet been determined.

In this study, we investigated the role of TAK1, TAB1 and TAB2 in LT-HSCs. We defined the capacity for long-term multipotency as well as the number of LT-HSCs in the competitively transplanted *Tak1-*, *Tab1-* and *Tab2*-deficient bone marrow cells. We also assessed whether the TNF pathway contributes to the failure of *Tak1-*deficient ST-HSC and LT-HSC maintenance by employing bone marrow cells with both *Tak1* and *Tnfr1* deficiency.

**Figure 1:**
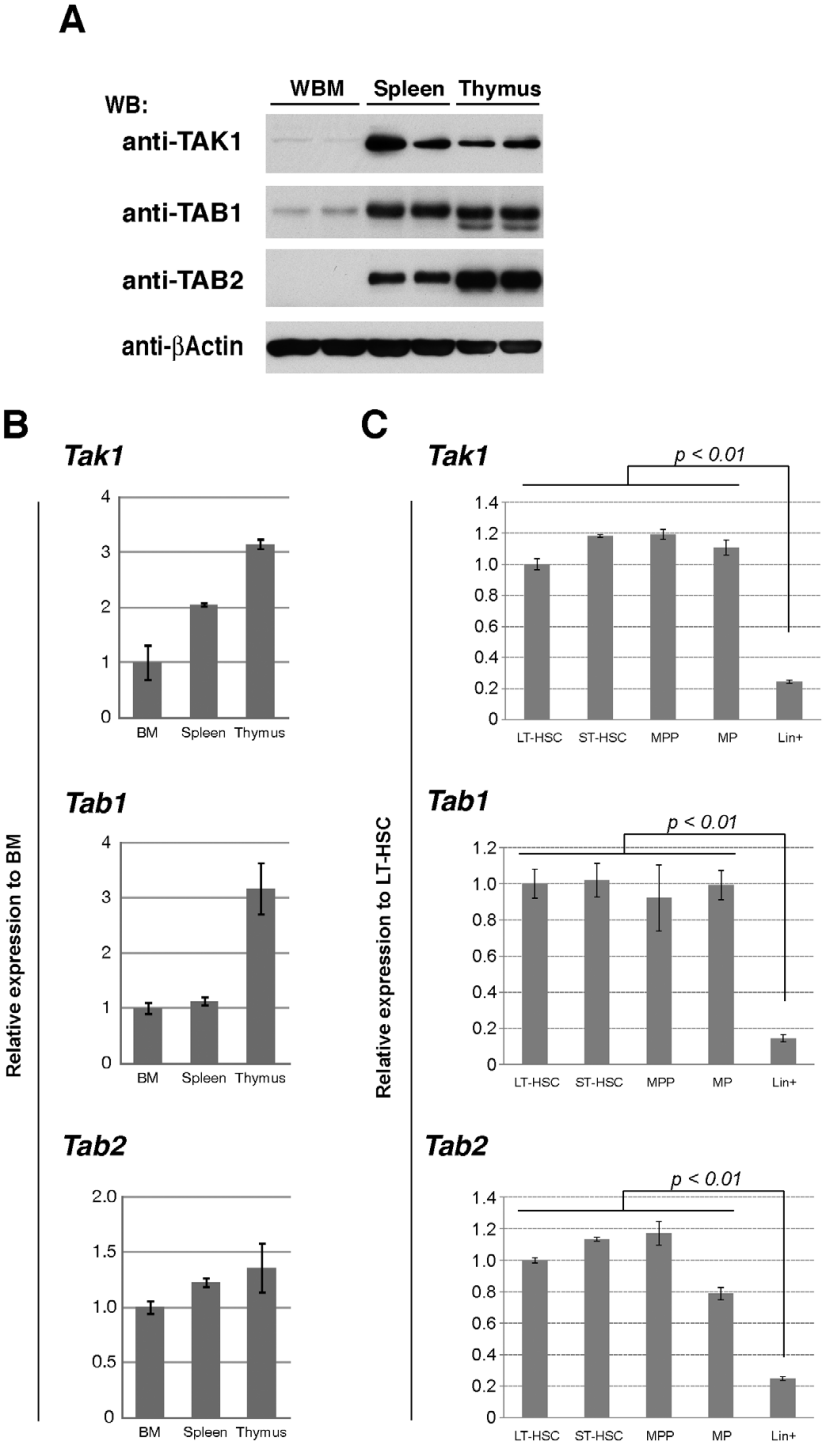
TAK1, TAB1 and TAB2 in bone marrow cells. (A) Expression of TAK1, TAB1 and TAB2 proteins. Whole cell extracts of the BMN cells (BM), splenocytes (Spleen) and thymocytes (Thymus) were analyzed by SDS-PAGE and Western blotted (WB) using the indicated antibodies. The amount of β-actin is shown as a loading control. (B) Expression of *Tak1*, *Tab1* and *Tab2* mRNA. Total RNA was isolated from BMN cells (BM), splenocytes (Spleen) and thymocytes (Thymus), and analyzed by qPCR. Expression level of each gene was normalized to that of *Actb* and shown as the relative value to BM. Data are presented as mean ± S.D. of three independent experiments. (C) Expression of *Tak1*, *Tab1* and *Tab2* mRNA. The cells in the LT-HSC (CD34^−^ Flt3^−^ LSK), ST-HSC (CD34^+^ Flt3^−^ LSK), MPP (CD34^+^ Flt3^+^ LSK), MP (Lineage^−^ c-Kit^+^ Sca-1^−^) or Lineage+ fractions from wild type mouse bone marrow were sorted by FACS, and total RNA was prepared. The relative amounts of *Tak1*, *Tab1*, *Tab2* and *Actb* mRNA were determined by qPCR. Expression level of each gene was normalized to that of *Actb* and shown as the relative value to LT-HSC. Data are presented as mean ± S.D. of four independent experiments.

## Materials and Methods

### Mice

*Tak1*-floxed (*Tak1^flox/flox^* or *Tak1^FF^*), *Tab1*-floxed (*Tab1^flox/flox^* or *Tab1^FF^*), *Tab2*-floxed (*Tab2^flox/flox^* or *Tab2^FF^*) mice were backcrossed for a minimum of five generations to C57BL/6 mice [20], [29], [30]. *Tnfr1*-deficient (*Tnfr1^−/−^*) (C57/BL6 backcrossed), *Rosa26-CreERT (*mixed background) mice were obtained from The Jackson Laboratory [31], [32]. This study was carried out in strict accordance with the recommendations in the Guide for the Care and Use of Laboratory Animals of the National Institutes of Health. All animal experiments were conducted with the approval of the North Carolina State University Institutional Animal Care and Use Committee (Approval number: 10-114B). All efforts were made to minimize animal suffering.

**Figure 2:**
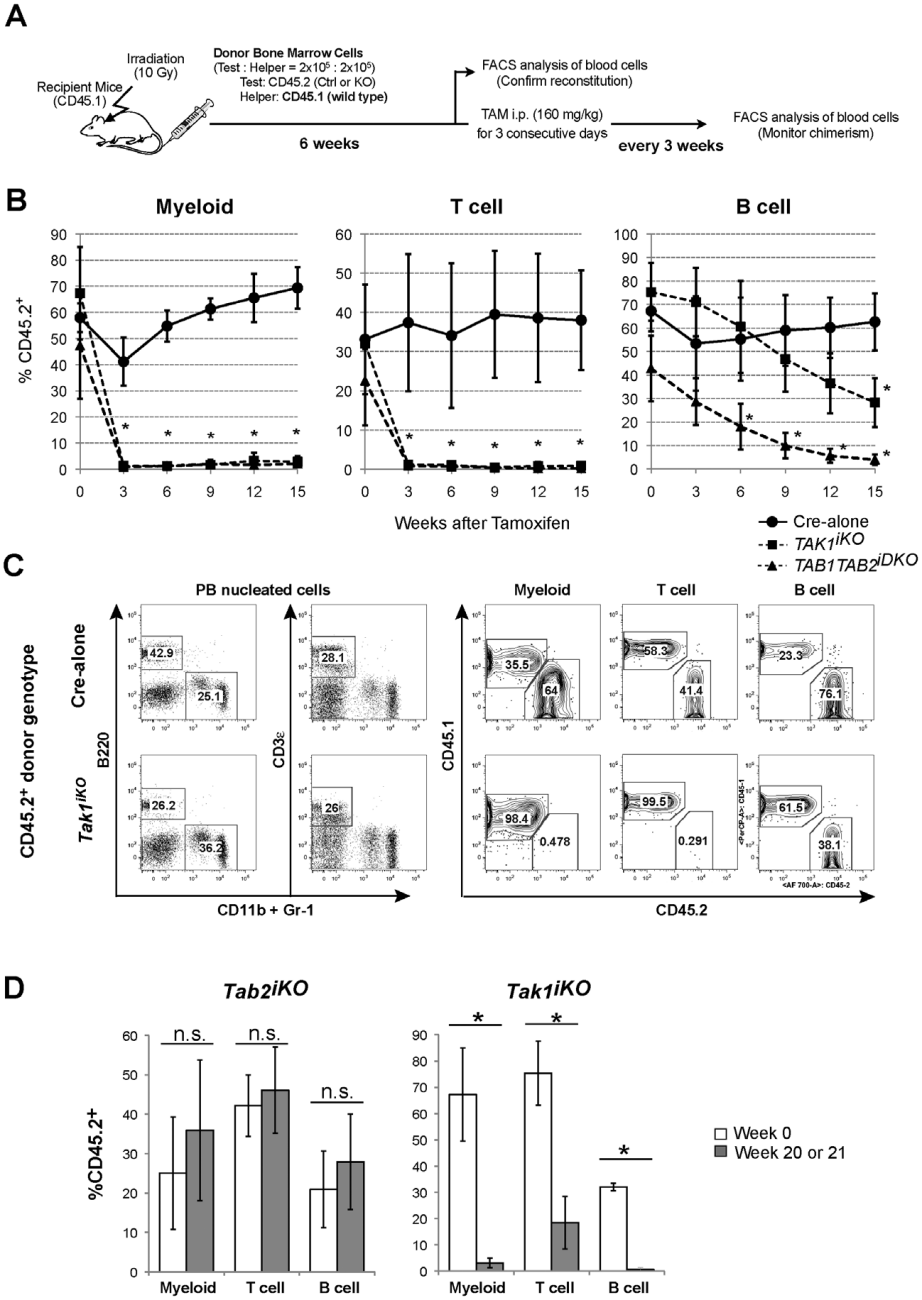
Competitive reconstitution assay. (A) Schematic representation of competitive transplantation. 2×10^5^ BMN cells from Cre-alone, *Tak1^iKO^* or *Tab1Tab2^iDKO^* mice (CD45.2^+^) were transplanted into lethally irradiated recipients (CD45.1) together with 2×10^5^ competitor wild type BMN cells (CD45.1^+^). At six weeks post-transplantation (designated as Week 0), the chimerism of myeloid, T and B cell populations in the recipients’ peripheral blood (PB) was analyzed, then the recipients were i.p. injected with tamoxifen at 160 mg/kg body weight for three consecutive days. (B) The chimerism of PB cell populations was monitored every three weeks, and shown as mean ± S.D. (*p<0.05). [Cre-alone (solid line, circles, n = 3), *Tak1^iKO^* (dashed line, squares, n = 3) or *Tab1Tab2^iDKO^* (dashed line, triangles, n = 4)]. (C) Representative flow cytometry data of blood cell chimerism. PB cells were collected 15 weeks after tamoxifen injection. Myeloid cells (CD11b^+^ or Gr-1^+^), B cells (B220^+^) and T cells (CD3ε^+^) in PB mononuclear cells were analyzed for the expression of CD45.1 or CD45.2. (D) Competitive reconstitution assay for *Tab2^iKO^* and *Tak1^iKO^* mice. Donor-derived chimerism of PBs was analyzed. The percentage of *Tab2^iKO^* or *Tak1^iKO^* PB myeloid, T and B cells (CD45.2^+^) in total PB myeloid, T and B cells (CD45.1^+^ and CD45.2^+^) before (Week 0, open bars) and 20 weeks or 21 weeks, respectively, after tamoxifen injection is shown (gray bars). Data are presented as mean ± S.D. (*p<0.05, n.s. not significant, n = 3).

### Competitive Reconstitution Assay

Seven- to nine-week-old C57BL6.SJL congenic mice were lethally irradiated (7–10 Gy) and intravenously infused with a mixture of bone marrow mononuclear (BMN) cells that were obtained from untreated test (CD45.2^+^) and competitor (CD45.1^+^) donor mice at five to six weeks of age. Each recipient received either 2×10^5^ test and 2×10^5^ competitor BMN cells (1∶ 1 ratio) or 1×10^6^ test and 2×10^5^ competitor BMN cells (5∶ 1) as indicated. At six to seven weeks post-transplantation, the peripheral blood (PB) samples from the recipient mice were analyzed to confirm successful reconstitution, and then mice were administered 160 mg/kg tamoxifen by intraperitoneal (i.p). injection for three consecutive days. PB samples were collected and analyzed every three weeks post-tamoxifen injection to determine the chimerism of circulating myeloid (CD11b^+^ or Gr-1^+^), B (B220^+^) and T (CD3ε^+^) cells.

**Figure 3:**
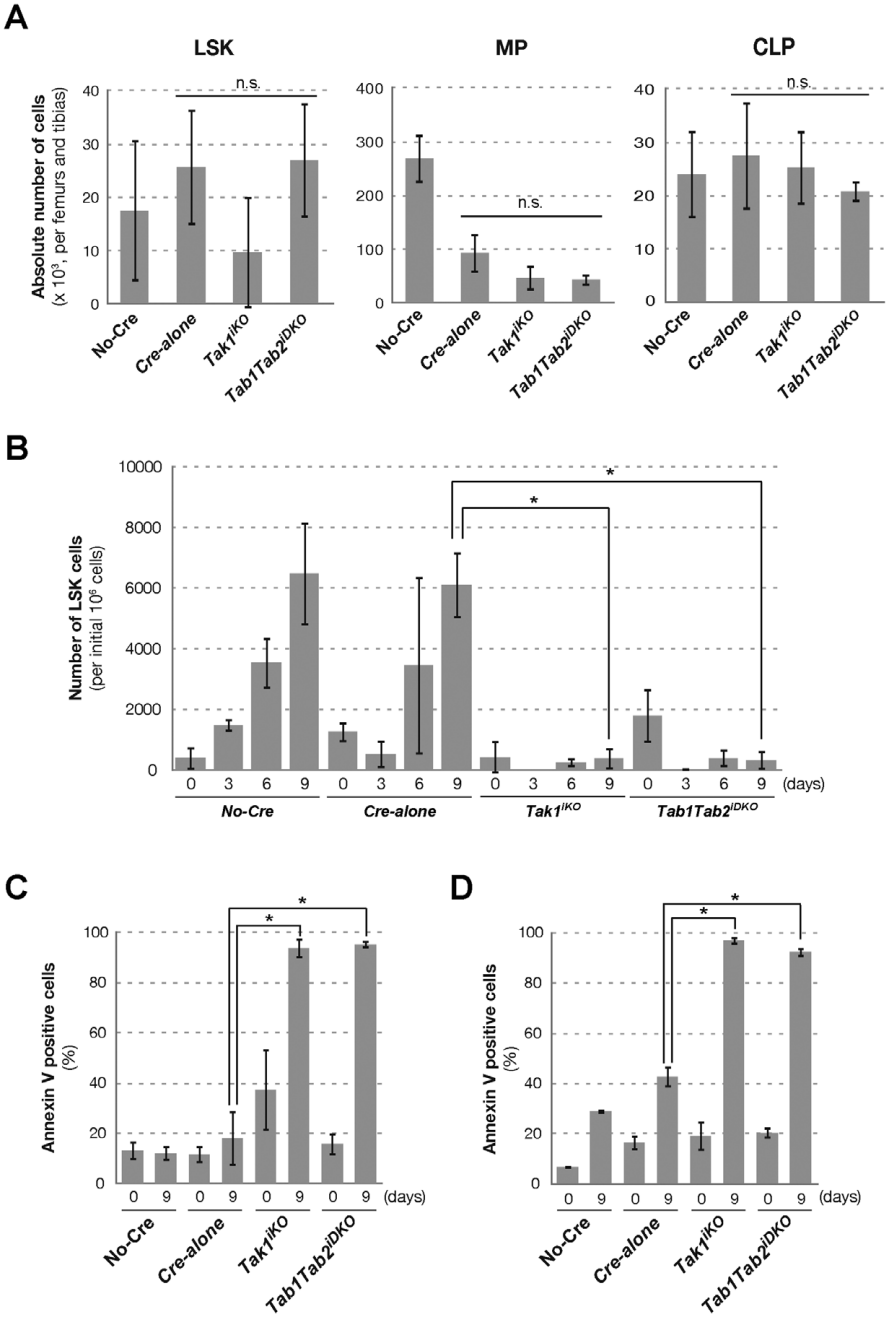
In vitro expansion of LSK population. (A) The mice with indicated genotypes were i.p. injected with tamoxifen (160 mg/kg) for three consecutive days and sacrificed at day 4. The total LSK, MP and CLP cell numbers in the femurs and tibias from each mouse were determined. Data are presented as mean ± S.D. (n.s. not significant, n = 3) (B) Whole BMN cells isolated at day 4 described in (A) were cultured in STIFA medium. At the time of plating, 2.5×10^6^ cells/well, 2×10^6^ cells/well and 1×10^6^ cells/well were plated for 3-, 6- and 9-day culture, respectively. Cells were harvested and analyzed by flow cytometry for lineage, Sca-1 and c-Kit surface markers. The absolute number of the LSK population in the harvested cells is shown as per 1×10^6^ initial BMN cells. Data are presented as mean ± S.D. (*p<0.05, n = 3) (C, D) Annexin V-binding assay of LSK (C) and lineage positive cells (D) after nine days of cell expansion in STIFA medium. Data are presented as mean ± S.D. (*p<0.05, n = 3).

### Flow Cytometric Analysis and Cell Sorting

Whole bone marrow cells were flushed from the femurs and tibias of six- to nine-week-old mice with Hank’s Balanced Salt Solution without magnesium and calcium [HBSS(–)]. The bone marrow cells were then suspended in 0.83% ammonium chloride (NH_4_Cl) for 5 min at room temperature to lyse red blood cells and washed with HBSS(–). BMN cells were resuspended in HBSS(–) and filtered through a 35-µm cell strainer (BD Biosciences) to obtain single cell suspension. The cells were incubated for 20 min on ice with anti-CD16/32 antibody to block FcγRII/III, followed by incubation with fluorochrome-conjugated antibodies against cell surface antigens as described below. After labeling, cells were washed once with HBSS(–), resuspended in HBSS(–) and analyzed on FACS LSRII (BD Biosciences). BMN cells or splenocytes were sorted using a FACSAria (BD Biosciences) or MoFlo (Beckman). Specific monoclonal antibodies against the following antigens were used for flow cytometric analysis: CD3ε (145-2C11), CD4 (RM4-5), CD8a (53-6.7), CD11b (M1/70), Gr-1 (RB6-8C5), CD19 (6D5), B220 (RA3-6B2), Ter-119 (TER-119), Sca-1 (E13-161.7), c-Kit (2B8), IL-7Rα (A7R34), CD34 (RAM34), CD41 (MWReg30), CD48 (HM48-1), CD150 (TC15-12F12.2), CD45.1 (A20), CD45.2 (104), and CD16/32 (93). A cocktail of monoclonal antibodies against CD4, CD8a, B220, CD19, CD11b, Gr-1 and Ter-119 was used as a lineage marker (Lineage). To collect PB samples, mice were bled from the right mandibular vein using a 5 mm animal lancet (Goldenrod, Mineola, NY), and the blood were collected into microcentrifuge tubes containing HBSS(–) buffer supplemented with EDTA at a final concentration of approximately 1 mM. Anticoagulated PB samples were treated with 0.83% NH_4_Cl, stained with antibodies against CD45.1, CD45.2, CD3ε, B220, CD11b and Gr-1, and analyzed on FACS LSRII (BD Biosciences).

**Figure 4:**
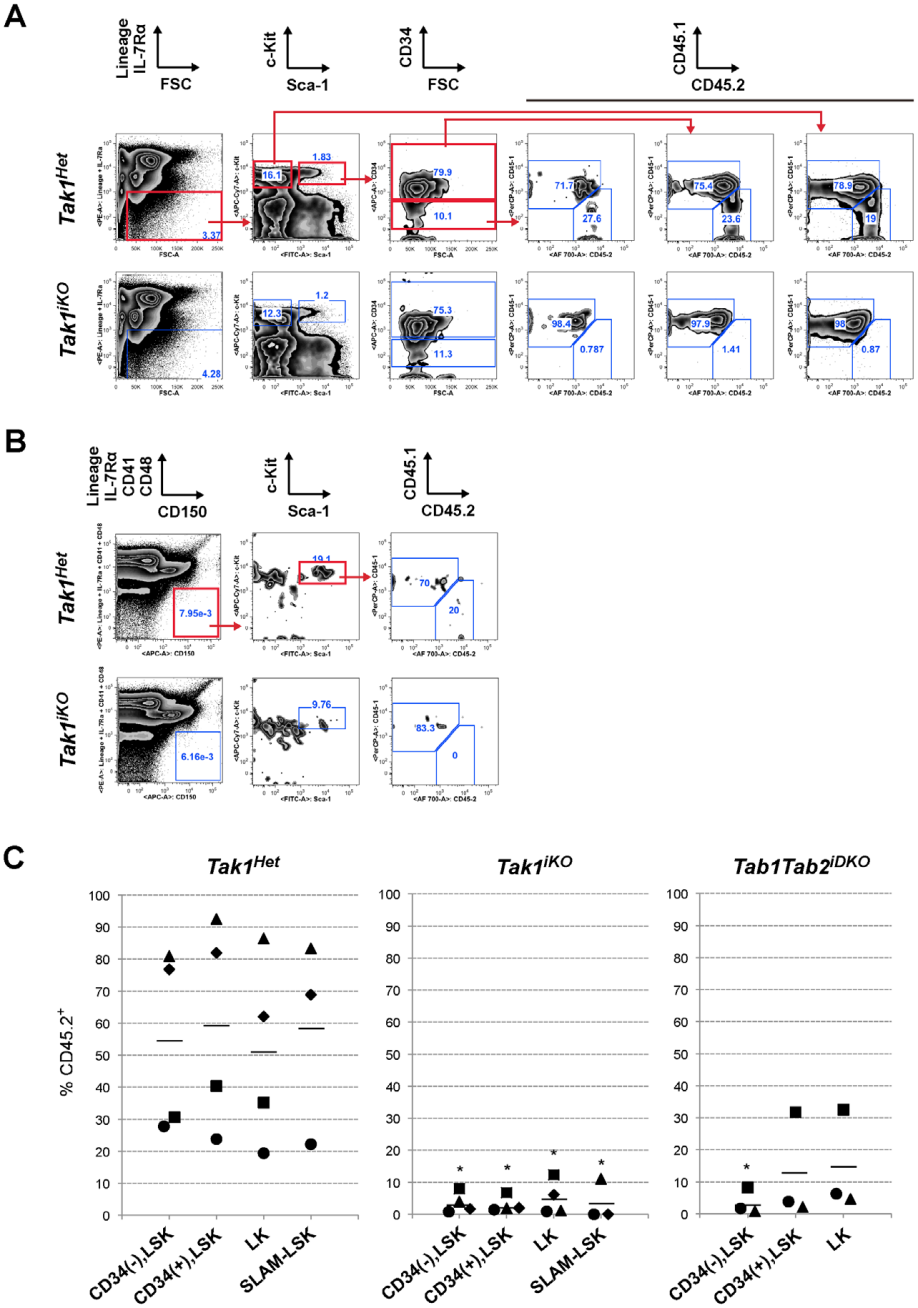
HSC chimerism. (A) The chimerism of LT-HSC, ST-HSC/MPP and MP in the recipient mice BM in competitive transplantation assay. The recipient mice (CD45.1^+^) were lethally irradiated and transplanted with a mixture of 1×10^6^ test (*Tak1^Het^* and *Tak1^iKO^*, CD45.2^+^) and 2×10^5^ competitor wild type (CD45.1^+^) BMN cells. Three weeks after tamoxifen injection, these recipients were analyzed for the chimerism of LT-HSC (CD34(–), LSK), ST-LSK/MPP (CD34(+) LSK) and MP (LK). Gating strategy and one result for each of the *Tak1^Het^* and *Tak1^iKO^* test BMN transplanted animals are shown. (B) Gating strategy for SLAM-LSK and one result for each of the *Tak1^Het^* and *Tak1^iKO^* test BMN transplanted animals are shown. (C) The chimerism of LT-HSC (CD34(–), LSK), ST-LSK/MPP (CD34(+) LSK), MP (LK) (n = 4) and SLAM-LSK (n = 3) in the recipient mice BM. The chimerism in each recipient is plotted, and the bars represent the average. * p<0.05 [*Tak1^Het^* versus *Tak1^iKO^* or *Tab1Tab2^iDKO^*].

### HPC Proliferation Assay

Mice were administered 160 mg/kg tamoxifen by i.p. injection for three consecutive days, sacrificed at day 4, and BMN cells were isolated. BMN cells were seeded into serum-free StemSpan SFEM medium (StemCell Technologies, Cat# 09600) supplemented with 10 mg/ml heparin (Calbiochem, Cat#375095), 100 µg/ml mSCF (Peprotech, Cat#250-03), 500 µg/ml mTPO (Peprotech, Cat#315-14), 100 µg/ml mIGF2 (R&D, Cat#792-MG), 100 µg/ml hFGF1 (Peprotech, Cat#100-17A), and 100 µg/ml mANGPTL3 (R&D, Cat#136-AN) [33] in 24-well plates; 2.5×10^6^ cells/well, 2×10^6^ cells/well and 1×10^6^ cells/well were plated for 3-, 6- and 9-day culture, respectively.

**Figure 5:**
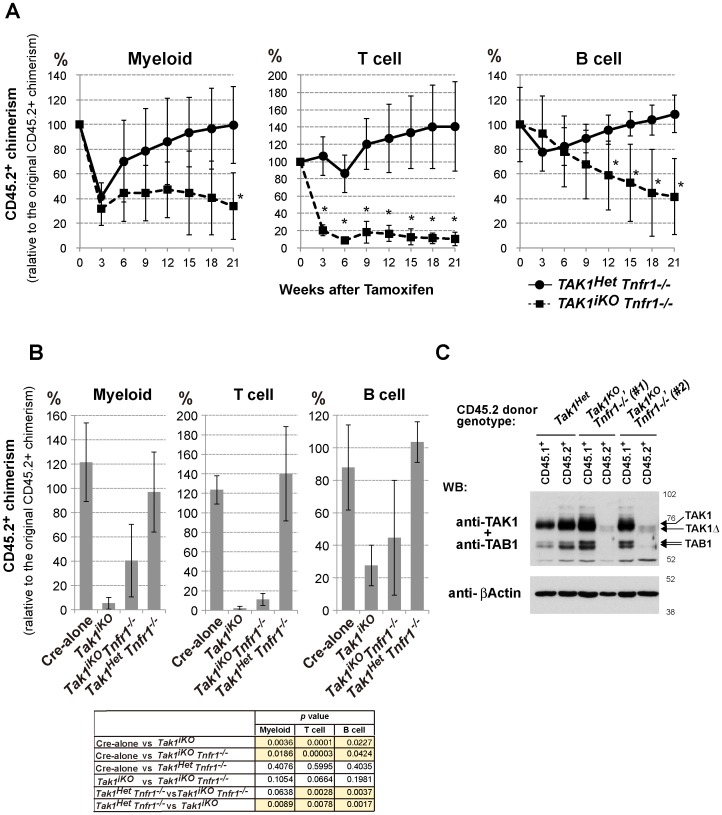
Ablation of TNF signaling partially restores the reconstitution potential of *Tak1*-deficient HSCs. (A) Competitive reconstitution assay. 2×10^5^ BMN cells from control *Tak1^Het^ Tnfr1^−/−^* (n = 3) or *Tak1^iKO^ Tnfr1^−/−^* mice (n = 4) (CD45.2^+^) were transplanted into lethally irradiated recipients (CD45.1^+^) together with 2×10^5^ competitor wild type BMN cells (CD45.1^+^). At six weeks post transplantation (designated as Week 0), the chimerism of myeloid, T and B cells in the recipients’ PB was analyzed, and then the recipients were i.p. injected with tamoxifen at 160 mg/kg body weight for three consecutive days. The chimerism of PB cells was monitored every three weeks, and is shown as the mean ± S.D. (*p<0.05) (B) Competitive reconstitution assay of Cre-alone (n = 3) and *Tak1^iKO^* (n = 3) was also performed as described above, and compared with *Tak1^Het^ Tnfr1^−/−^* (n = 3) and *Tak1^iKO^ Tnfr1^−/−^* (n = 4) at 18 weeks post-tamoxifen injection. The table shown below the graphs indicates statistical significance for the indicated comparisons. *P* values of less than 0.05 are highlighted. (C) Expression of TAK1 and TAB1 proteins in the donor-derived splenocytes. Whole spleen cells from control *Tak1^Het^* and two independent *Tak1^iKO^ Tnfr1^−/−^* transplanted mice (#1 and #2) at 15 weeks post-tamoxifen injection were sorted into the CD45.1+ or CD45.2+ population. Total cell lysates from the sorted splenocytes from control *Tak1^Het^*, and *Tak1^iKO^ Tnfr1^−/−^* #1 and #2 were analyzed by SDS-PAGE and Western blotted with anti-TAK1, TAB1 or anti-β-actin antibodies. The positions of molecular weight markers are shown on the right. The arrows indicate the bands corresponding to endogenous TAK1 and TAB1, and truncated TAK1 (TAK1Δ) resulting from Cre-mediated recombination.

### Quantitative Real Time PCR Analysis

For gene expression analysis, LT-HSC (CD34^−^, Flt3^−^, LSK), ST-HSC (CD34^+^, Flt3^−^, LSK), MPP (CD34^+^, Flt3^+^, LSK), MP (Lineage^−^, c-Kit^+^, Sca-1^−^) and Lineage^+^ cells were sorted from WBM cells using a FACSAria cell sorter. Total RNA was isolated from these cells using an RNeasy kit (QIAGEN) and transcribed into cDNA using SuperScript VILO cDNA Synthesis Kit (Life Technologies). Expression levels of *Tak1, Tab1 and Tab2* were determined by quantitative real time PCR (qPCR) and normalized to the level of *Actb*. The following primers were used: *Tak1*-forward, 5′-CGTCTTCTGCCAGTGAGATG-3′; *Tak1*-reverse, 5′-ATCTTTTGCTCTCCACTTAGCTT-3′; *Tab1*-forward, 5′-ACCCTGCTGGTGAGGAACT-3′; *Tab1*-reverse, 5′-AGGGACAGAGTCACACTAGTCTT-3′; *Tab2*-forward, 5′-GGATAGAATAAGCGAAGCCCGGAA-3′; *Tab2*-reverse, 5′-CTCTTTGAAGCCGTTCCATCCT-3′; *Actb*-forward, 5′- CATCCGTAAAGACCTCTATGCCAAC-3′; and *Actb*-reverse, 5′-ATGGAGCCACCGATCCACA-3′. Data are shown as values relative to LT-HSCs.

**Figure 6:**
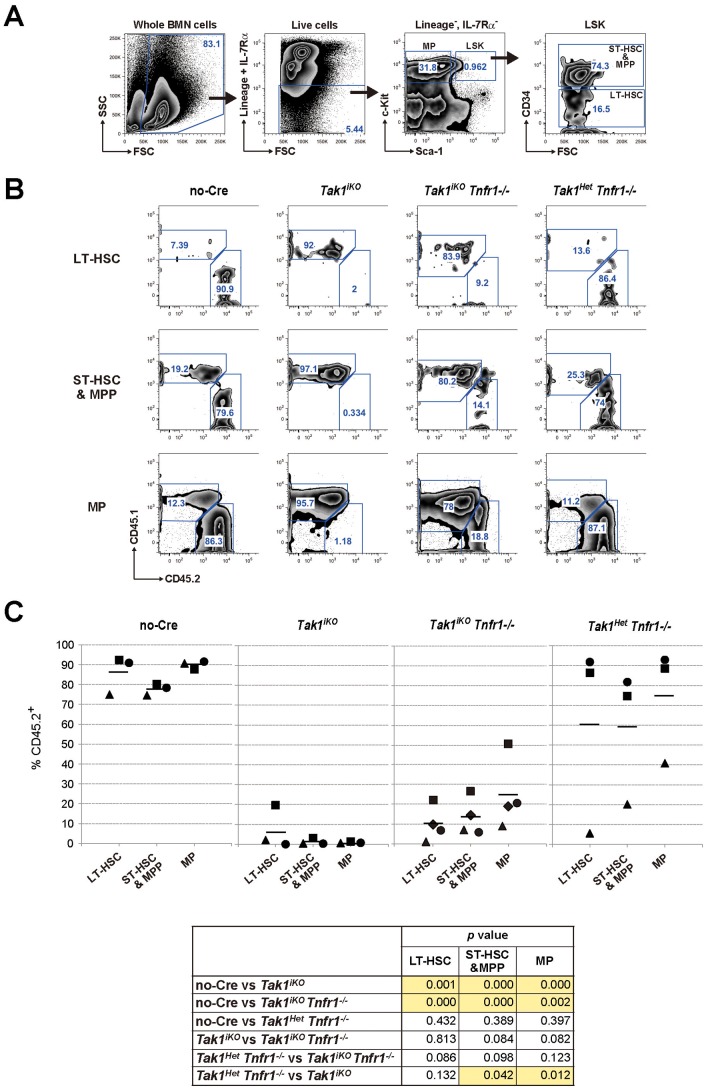
Impaired LT-HSC function by *Tak1* deficiency cannot be rescued by ablation of TNF signaling. (A) Gating strategy for LT-HSC, ST-HSC/MPP and MP in the competitive reconstitution assay. (B) Representative results of chimerism analysis of No-Cre, *Tak1^iKO^*, *Tak1^iKO^ Tnfr1^−/−^*, and *Tak1^Het^ Tnfr1^−/−^* test donor transplanted mice are shown. (C) The chimerism of LT-HSC, ST-HSC/MPP and MP in the recipient mouse BM. Lethally irradiated recipient mice (CD45.1^+^) were transplanted with a mixture of 5×10^5^ test (controls, No-Cre and *Tak1^Het^ Tnfr1^−/−^*; and *Tak1* deficient, *Tak1^iKO^* and *Tak1^iKO^ Tnfr1^−/−^*, CD45.2^+^) and 5×10^5^ competitor wild type (CD45.1^+^) BMN cells. The recipients were i.p. injected with tamoxifen at 160 mg/kg body weight for three consecutive days starting at six weeks post transplantation. Twenty-two weeks or more after tamoxifen injection, recipients were analyzed for the chimerism of LT-HSC (CD34^−^, LSK), ST-HSC/MPP (CD34^+^, LSK) and MP (LK) in BMN cells. The chimerism in each recipient is plotted, and the bars represent the average. The table shown below the graphs indicates statistical significance for the indicated comparisons. P values of less than 0.05 are highlighted. Mice were i.p. injected with tamoxifen at 160 mg/kg body for three consecutive days (*Tak1^iKO^, Tab1^iKO^, Tab2^iKO^* and *Tab1Tab2^iDKO^*) or untreated (*Tak1FF, Tak1FF Cre* or *Tab1Tab2FF*) and sacrificed on day 4. Genomic DNA isolated from BMN cells was analyzed by qPCR using primers designed to detect a portion of the genome flanked by two loxP sites to determine the relative copy number of intact *Tak1*, *Tab1* or *Tab2* genome. Data are presented as mean ± S.D. of three independent experiments. Mice with the indicated genotype were i.p. injected with tamoxifen (160 mg/kg) for three consecutive days, and splenocytes were collected at Day 14 (left panels) or Day 4 (right panels). Whole cell extracts were prepared and analyzed by Western blot using the indicated antibodies. Asterisks indicate non-specific bands. Mice with the indicated genotype were i.p. injected with tamoxifen (160 mg/kg body weight) for three consecutive days, and BMN cells were collected at Day 4. (A) The number of total BMN cells of femurs and tibias were counted on hemacytometer. The cell number in MP or LSK population was determined by FACS analysis. Data are presented as mean ± S.D. (n = 3) (*p<0.05) (B) Representative FACS plots for Sca-1 vs c-Kit in lineage-negative population of BMN cells are shown. The gates for MP and LSK and percentages of each population are indicated. (A) 2×10^5^ BMN cells from *Tab1^FF^*, *Tab1^iKO^*, *Tab2^FF^*, or *Tab2^iKO^* mice (CD45.2^+^) were transplanted into lethally irradiated recipients (CD45.1^+^) together with 2×10^5^ competitor wild type BMN cells (CD45.1^+^). At six weeks post transplantation, the chimerism of myeloid, T and B cells in the recipients’ PB was analyzed, then the recipients were i.p. injected with tamoxifen (160 mg/kg body weight) for three consecutive days. The chimerism of PB cells was monitored every three weeks. In each experiment, a CD45.1/CD45.2 mixture of BMN cells was transplanted into two recipients, and the average of their blood cell chimerism is shown. (B) Splenocytes from control No-Cre or *Tab2^iKO^* transplanted mice (#1 and #2) at 21 weeks post-tamoxifen injection were sorted into the CD45.1^+^ or CD45.2^+^ population. Total cell lysates from the sorted splenocytes were analyzed by SDS-PAGE and Western blotted with indicated antibodies. The positions of molecular weight markers are shown on the right. The asterisks indicate the non-specific bands. Mice with the indicated genotype (the same animals used for the *in vitro* LSK expansion assay in Figure 3) were i.p. injected with tamoxifen (160 mg/kg body weight) for three consecutive days, and BMN cells were collected at Day 4. BMN cells were analyzed by FACS to determine the number of B cell (B220^+^), T cell (CD3ε^+^) and granulocyte (CD11b^+^ and Gr-1^+^) per femurs and tibias. Data are presented as mean ± S.D (n = 3).

For the verification of gene deletion, genomic DNA was isolated from BMN cells using a DNeasy Blood & Tissue Kit (QIAGEN) according to the manufacturer’s instructions. Using qPCR, the relative copy number was determined by generating a standard curve. The following primers were used: *Tak1*-forward, 5′-AGGTTGTCGGAAGAGGAGCT-3′; Tak1-reverse, 5′-CTCCACAATGAAAGCCTTCC-3′; Tab1-forward, 5′-ACCCTGTTTCTGTGCCCTACTCAA-3′; Tab1-reverse, 5′-ACTGTGAGAGCCGTTGACCATCT-3′; Tab2-forward, 5′-TGCGCTGTTCTCTCTCAGGA-3′; and Tab2-reverse, 5′-CAAGTCCAAGTTGAGAGAAG-3′. Data are shown as values relative to *Tak1^flox/flox^* BMN cells.

### Western Blotting

BMN cells, splenocytes or thymocytes were lysed in extraction buffer containing 20 mM HEPES (pH7.4), 150 mM NaCl, 1.5 mM MgCl_2_, 0.5 mM DTT and 0.5% Triton X-100 supplemented with a protease inhibitor cocktail (ThermoScientific) and phosphatase inhibitor cocktail (Nacalai Tesque). Cell debris and nuclei were pelleted by centrifugation at 20,000×g for 10 min at 4°C, and the resulting supernatant was used for Western blotting analysis.

### Statistical Analysis

Data were analyzed using two-tailed Student’s *t* test. Values are expressed as means ± S.D. Differences were considered significant at *p*<0.05.

## Results and Discussion

### The TAK1 Complex is Required for LT-HSC Function

To determine the importance of the TAK1 complex in HSCs, we first examined the protein levels of TAK1, TAB1 and TAB2 in bone marrow cells (Figure 1A). To our surprise, protein levels of TAK1, TAB1 and TAB2 were all very low in bone marrow cells, when compared to the levels in the spleen and thymus. We also found that the levels of mRNA of *Tak1*, *Tab1* and *Tab2* were also lower in bone marrow cells, but the differences among the organs were much less pronounced, compared to the differences in the protein levels (Figure 1B). These protein levels may also be post-transcriptionally modulated. Among bone marrow cells, the mRNA levels of *Tak1*, *Tab1* and *Tab2* were significantly higher in the undifferentiated populations including LT-HSCs and progenitor cells compared to differentiated bone marrow cells (Figure 1C). This raises the possibility that TAK1, TAB1 and TAB2 may play an important role in HSCs. Therefore, we characterized bone marrow cells in adult mice having deletions of *Tak1*, *Tab1* or *Tab2* genes using the ubiquitously-expressed inducible Cre recombinase system, *Rosa26.CreERT* deleter mice [31]. *Rosa26.CreERT Tak1^flox/flox^* (referred to as *Tak1^iKO^*), *Rosa26.CreERT Tab1^flox/flox^* (referred to as *Tab1^iKO^*), and *Rosa26.CreERT Tab2^flox/flox^* (referred to as *Tab2^iKO^*) were compared with littermate or age-matched controls including *Rosa26.CreERT Tak1^flox/+^* (referred to as *Tak1^Het^* or het), *Rosa26.CreERT Tab1^flox/+^* (referred to as *Tab1^Het^* or het), *Rosa26.CreERT Tab2^flox/+^* (referred to as *Tab2^Het^* or het), no-Cre (*Tak1^flox/flox^*, *Tab1^flox/flox^* and *Tab2^flox/flox^*) and Cre-alone (*Rosa26.CreERT*). Tamoxifen was intraperitoneally injected once per day for three consecutive days (the first day of tamoxifen injection is designated as day 1). Intact *Tak1*, *Tab1* and *Tab2* genes were greatly decreased in *Tak1^iKO^*, *Tab1^iKO^* and *Tab2^iKO^* in bone marrow cells at day 4 (Figure S1), indicating that these genes were efficiently deleted four days after the start of tamoxifen treatment. Since *Tak1* deficiency causes damage to multiple tissues within four days, which will be reported elsewhere, the reduction in the protein levels was confirmed by immunoblots of TAB1 and TAB2 in splenocytes at day 4 (Figure S2). We note here that ubiquitous expression of Cre in the *Rosa26.CreERT* mice including hets and Cre-alone, caused weight loss at days 5-7, which is presumably associated with toxic effects of Cre expression [34]. The number of whole bone marrow cells and lineage negative Sca1^-^ c-Kit^+^ myeloid progenitor (MP) cells was diminished at day 4 (Figure S3A and B), as previously reported [35]. In contrast, the number of LSK cells was not noticeably decreased by Cre expression (Figure S3A and B). Thus, although Cre exerted significant effects on bone marrow cells, this system might still be suitable for examining the effects of gene deletions in HSCs. Nonetheless, to exclude any effects of Cre toxicity, we used Cre-expressing controls, Cre-alone or hets, in all experiments.

To examine whether TAK1, TAB1 and TAB2 are involved in HSC function in vivo, we determined long-term chimerism in the competitive transplantation assay. *Tak1^iKO^*, *Tab1^iKO^*, *Tab2^iKO^*, *Tab1Tab2^iDKO^*, hets, Cre-alone and no-Cre control bone marrow cells, which express CD45.2, were mixed with CD45.1^+^ wild type competitors at a 1∶1 ratio (and 5∶1 ratio in some experiments as indicated in the figure legends) and were transplanted into lethally irradiated CD45.1^+^ recipients. After engraftment of CD45.2^+^ hematopoietic cells was confirmed, tamoxifen was injected, and myeloid, T and B cells in peripheral blood were analyzed (see a schematic drawing in Figure 2A). To monitor the reconstitution potential of HSCs, chimerism was monitored every three weeks for a 15-week period. Bone marrow from control including Cre alone and hets exhibited the same trend of chimerism; a transient decrease in myeloid and B-cell chimerism of Cre-alone and hets cells was observed around three weeks post tamoxifen injection (Figure 2B). However; chimerism was restored to the original levels by 6–12-weeks post-tamoxifen injection. Thus, Cre expression alone reduces myeloid cells and/or their progenitors as shown in Figure S3, but does not have a major impact on HSC function and has no impact on LT-HSC function.

In contrast, deletion of *Tak1* sharply diminished the myeloid and T-cell populations and gradually reduced the B-cell population (Figures 2B-D). Since myeloid cells have a short life-span, this sharp drop of cell number is likely to be the result of MP and/or HSC impairment due to *Tak1* deletion. In contrast to myeloid cells, T cells are known to live for several weeks to months. Therefore, the common lymphoid progenitor (CLP) and/or HSC impairment by *Tak1* deletion cannot account for the sharp drop of the peripheral T-cell population. It has previously been reported that TAK1 is essential for T-cell survival using a T cell-specific *Tak1* deletion [36]. Therefore, the depletion of *Tak1*-deficient T cells in the peripheral blood is likely due to reduced survival. B-cell chimerism was gradually but constantly decreased after tamoxifen injection. This observation suggests that B cells do not require TAK1 for their survival, which is consistent with the findings of an earlier study [30]. This gradual decline indicates that HSC function is impaired by deletion of *Tak1*. Collectively, these results suggest that TAK1 is essential for repopulating potential of LT-HSC, and in addition to that, TAK1 is important for T-cell survival in the peripheral blood.

While *Tak1* deletion donor derived cells were almost completely depleted at 21 weeks, *Tab2* single deletion donor-derived cells exhibited repopulating ability for more than 20 weeks post-tamoxifen treatment, (Figure 2D and Figure S4A). Tamoxifen-induced gene deletion was confirmed by immunoblotting of splenocytes from *Tab2^iKO^* bone marrow chimera mice at 21 weeks post-tamoxifen injection (Figure S4B). TAB2 protein was not detectable in the CD45.2^+^ population even after 21 weeks, indicating that TAB2 is largely dispensable for HSC function. *Tab1* single deletion donor bone marrow cells also exhibited ability to repopulate, although these modified cells were slightly less efficient compared to “no-Cre” controls (Figure S4A). Thus, either TAB1 or TAB2 is not essential for HSC function. Since we have recently found that TAB1 and TAB2 are functionally redundant in the activation of TAK1 in the epidermis and the intestinal epithelium [22], we further generated *Rosa26.CreERT Tab1^flox/flox^ Tab2^flox/flox^* (referred to as *Tab1Tab2^iDKO^*) mice and performed the competitive transplantation assay. In contrast to *Tab1* or *Tab2* single deletion, double deletion of *Tab1* and *Tab2* generated the identical chimerism pattern produced by *Tak1* deletion (Figure 2B). Thus, TAB1 and TAB2 redundantly function to activate TAK1 and together play an indispensable role in the reconstitution activity of HSC. TAB1 is important for stress-induced activation of TAK1 [21], while TAB2 functions as an adaptor for cytokine receptor signaling pathways [15], [19]. Bone marrow cells are constantly exposed to stressors such as reactive oxygen species [8] and cytokines such as TNF [37]. Thus, it is likely that both stressors and cytokines activate TAK1 through TAB1 and TAB2 in bone marrow under normal conditions. Because stressors and cytokines potentially damage HSCs, we assume that stressor- and cytokine-induced activation of TAK1 is required for HSC maintenance.

### TAK1 is Important for LSK Expansion

Reconstitution potential of HSCs was abolished by *Tak1* or *Tab1 Tab2* double deletion as shown in Figure 2B. This suggests that ablation of TAK1 signaling impairs either ability of differentiation, expansion or survival of HSCs. If only the capacity for differentiation is impaired, then *Tak1^iKO^* and *Tab1Tab2^iDKO^* HSCs should still be able to expand in the in vitro expansion assay. Accordingly, we treated *Tak1^iKO^* and *Tab1Tab2^iDKO^* and control mice with tamoxifen in vivo for three consecutive days, and at the next day, bone marrow cells were isolated. At day 4, LSK cells were found to be largely viable, and LSK population number was not significantly reduced in *Tak1^iKO^* and *Tab1Tab2^iDKO^* (Figure 3A). MP, CLPs, B and T cells and granulocytes were not reduced by *Tak1* or *Tab1 Tab2* double deficiency at this point (Figure 3A and Figure S5). To test the capacity for expansion, the isolated bone marrow cells were plated in StemSpan serum-free medium containing the growth factors SCF, TPO, IGF-2, FGF-1 and Angptl3 (STIFA medium) [33] (Figure 3B). As expected, the number of LSK cells was increased in control no-Cre and Cre*-*alone bone marrow cells, while *Tak1^iKO^* and *Tab1Tab2^iDKO^* LSK cells failed to expand. Annexin V-binding, which is associated with apoptosis, was greatly increased at day 9 of the expansion assay in both the *Tak1^iKO^* and *Tab1Tab2^iDKO^* LSK fractions (Figure 3C). Differentiated lineage positive *Tak1^iKO^* and *Tab1Tab2^iDKO^* cells were also found to be largely annexin V binding positive at day 9 (Figure 3D). These results indicate that deficiency of *Tak1* impairs LSK expansion due to increased apoptosis, raising the possibility that TAK1 signaling may be required for survival of HSCs.

### Ablation of TAK1 Signaling Depletes Long-term HSCs

Since LT-HSCs have been reported to double approximately every 36 days (activated HSCs) or 145 days (dormant HSCs) in mice [38], and the number of LT-HSCs is less than 0.001% of bone marrow cells, the in vitro expansion assay could not permit the determination of whether the ablation of TAK1 signaling caused the failure of expansion or death of LT-HSCs. To further assess TAK1 regulation of LT-HSCs, we determined the populations of LT-HSCs at a relatively early time point after gene deletion in the competitive transplantation assay. If the TAK1 complex regulates self-renewal but not survival of LT-HSCs, then *Tak1^iKO^* LT-HSCs should still be present at three weeks post-gene deletion. Therefore, we analyzed bone marrow cells three weeks after tamoxifen injection in the competitively transplanted *Tak1^iKO^* bone marrow chimeric mice. While control *Tak1^Het^* bone marrow cells reconstituted CD45.2^+^ LT-HSC and ST-HSC/MPP populations with a reasonable level of chimerism of 30–90%, only small numbers of *Tak1^iKO^* CD45.2^+^ LT-HSC and ST-HSC/MPP cell numbers were present in in the competitively transplanted *Tak1^iKO^* bone marrow chimeric mice (Figures 4A, gating strategy, and 4C). Additionally, LSK population with SLAM family receptor CD150^+^, which is another phenotypic marker of LT-HSCs [5], was also diminished in the *Tak1^iKO^* CD45.2^+^ population (Figures 4B, gating strategy, and 4C). Similar to *Tak1^iKO^* donor-derived LSKs, both of the *Tab1Tab2^iDKO^* donor-derived LT-HSCs were also eliminated within three weeks post-tamoxifen injection (Figure 4C). In aggregate, these data suggest that TAB1/TAB2-dependent TAK1 signaling is required for survival of LT-HSCs.

### *Tnfr1* Deficiency Partially Rescues the Failure of *Tak1*-deficient HSC Function

TNF receptor deficiency is reported to partially restore the reconstitution ability of *Tak1*-deficient bone marrow cells [27]. *Tak1*-deficiency is known to sensitize cells to TNF-induced cell death without altering TNF production in epithelial tissues [25], [26]. Therefore, *Tak1*-deficient HSCs may be killed by TNF which is expressed at a constant level in the bone marrow. Since the earlier study in *Tak1*-deficient bone marrow cells focused on relatively short-term reconstitution assays and did not provide detailed analyses for the capacity of *Tak1* and TNF receptor double deficient HSCs for reconstitution, the contribution of TNF signaling to the inability of *Tak1*-deficient HSCs to reconstitute the hematopoietic system is not fully defined. Thus, we next investigated the reconstitution potential of *Tak1^iKO^* bone marrow cells in a *Tnfr1^−/−^* background over both a short and long term. The short-term reconstitution reflects ST-HSC function, while the long-term reconstitution requires LT-HSC function. Since TNF signaling is not known to profoundly impact on HSC function [37], we compared *Tak1^iKO^ Tnfr1^−/−^* with control *Tak1^Het^ Tnfr1^−/−^* and also with *Tak1^iKO^* cells. The *Tak1^iKO^ Tnfr1^−/−^* and control bone marrow cells were subjected to a competitive transplantation assay using the same procedure as described in Figure 2A. The reconstitution from donor-derived cells was analyzed for 18 weeks after tamoxifen injection (Figure 5A). The level of reconstitution from Cre-positive control (*Tak1^Het^ Tnfr1^−/−^*) donor cell was transiently reduced at three weeks (myeloid and B cells) and at six weeks (T cells) post-tamoxifen injection, which is associated with Cre toxicity, as described above. The transient reduction in control test donor cells was restored by six to nine weeks, and the controls exhibited stable reconstitution thereafter. In contrast, *Tak1^iKO^ Tnfr1^−/−^* T cells were significantly reduced within three weeks and remained at a low level, and *Tak1^iKO^ Tnfr1^−/−^* myeloid cells were also reduced, but without statistical significance until 21 weeks post-treatment (Figure 5A). The level of *Tak1^iKO^ Tnfr1^−/−^* B-cell reconstitution slowly but persistently declined over 18 weeks, which resembles the *Tak1^iKO^* phenotype shown in Figure 2B. Thus, *Tak1* deficiency still impaired HSC function even in the absence of TNFR1 signaling. However, the reconstitution level of *Tak1^iKO^* test donor at 18 weeks was found to be lower than that of *Tak1^iKO^ Tnfr1^−/−^* (Figure 5B), suggesting that *Tnfr1* deficiency might partially rescue the ST-HSC function in *Tak1^iKO^*. To confirm the gene deletion, the TAK1 protein in splenocytes from two independent *Tak1^iKO^ Tnfr1^−/−^* donor mice (#1 and #2) and control mice was analyzed at 15 weeks (Figure 5C and Figure S6). Test donor- and competitor-derived splenocytes were separated by FACS, and immunoblotting of TAK1 and TAB1 was performed. In both *Tak1^iKO^ Tnfr1^−/−^* donor transplanted mice, we could collect live test donor-derived splenocytes as shown in Figure S6, although #2 exhibited a lower test donor-derived cell number. The *Tak1^iKO^ Tnfr1^−/−^* donor-derived splenocytes exhibited almost no expression of TAK1 (Figure 5C). TAB1 was also found to be greatly reduced in *Tak1^iKO^ Tnfr1^−/−^* splenocytes (Figure 5C), as in other *Tak1*-deficient cell types [22]. Thus, TAK1 is not absolutely required for at least the short-term reconstitution of the hematopoietic system if the *Tnfr1* gene is deleted. These results suggest that *Tak1^iKO^ Tnfr1^−/−^* ST-HSCs are at least in part functionally intact and can reconstitute the hematopoietic system in the short term.

### TAK1 is Required for HSC Maintenance Independently of TNF-induced Cell Death

*Tak1^iKO^ Tnfr1^−/−^* bone marrow cells could reconstitute the hematopoietic system to some extent for at least 18 weeks. However, all *Tak1^iKO^ Tnfr1^−/−^* derived myeloid, T- and B-cell chimerism levels were low and gradually declined, while control chimerism was not altered for a long time (Figure 5A). This phenomenon might indicate impaired LT-HSC function in *Tak1^iKO^ Tnfr1^−/−^* bone marrow cells. To investigate this possibility, we analyzed bone marrow cells from competitively transplanted mice more than 22 weeks post *Tak1* gene deletion (Figure 6). We phenotypically determined LT-HSC, ST-HSC/MPP and myeloid progenitor (MP) (Figure 6A, gating strategy). Controls including those receiving no-Cre and *Tak1^Het^ Tnfr1^−/−^* test donor cells exhibited a chimerism level of 70–90% (Figure 6B-C) except one animal receiving *Tak1^Het^ Tnfr1^−/−^* cells. *Tak1^iKO^* ST-HSC/MPP and MP cells were completely depleted as expected, and *Tak1^iKO^ Tnfr1^−/−^* ST-HSC/MPP and MP cells were also reduced (Figure 6B-C). However, the level of *Tak1^iKO^ Tnfr1^−/−^* MP cells was higher than *Tak1^iKO^* MP cells, suggesting that *Tnfr1* deficiency might have partially rescued MPs. In contrast, *Tak1^iKO^ Tnfr1^−/−^* LT-HSC and ST-HSC/MPP cells were still significantly reduced compared to the controls. Thus, TAK1 is required for the maintenance of the HSC pool, which is independent of the prevention of TNF-induced cell death.

Our results demonstrate that TAK1 protects the hematopoietic system through two distinct mechanisms: one is by the prevention of TNF-induced cell death, which is important for the maintenance of ST-HSCs, and the second is via a TNF-independent process that contributes to LT-HSC maintenance. Undoubtedly, our understanding of HSC homeostasis will be enhanced by further study defining the TNF-independent mechanism through which TAK1 signaling regulates the LT-HSC compartment.

## Supporting Information

Figure S1 **Gene deletions of** ***Tak1*** **,** ***Tab1*** **and** ***Tab2*** **in BMN cells.** Mice were i.p. injected with tamoxifen at 160 mg/kg body for three consecutive days (*Tak1^iKO^, Tab1^iKO^, Tab2^iKO^* and *Tab1Tab2^iDKO^*) or untreated (*Tak1FF, Tak1FF Cre* or *Tab1Tab2FF*) and sacrificed on day 4. Genomic DNA isolated from BMN cells was analyzed by qPCR using primers designed to detect a portion of the genome flanked by two loxP sites to determine the relative copy number of intact *Tak1*, *Tab1* or *Tab2* genome. Data are presented as mean ± S.D. of three independent experiments.
(TIF)

Figure S2 **Deletion of TAB1 and TAB2 in splenocytes.** Mice with the indicated genotype were i.p. injected with tamoxifen (160 mg/kg) for three consecutive days, and splenocytes were collected at Day 14 (left panels) or Day 4 (right panels). Whole cell extracts were prepared and analyzed by Western blot using the indicated antibodies. Asterisks indicate non-specific bands.
(TIF)

Figure S3 **Effect of** ***CreERT*** **activation on hematopoietic cells.** Mice with the indicated genotype were i.p. injected with tamoxifen (160 mg/kg body weight) for three consecutive days, and BMN cells were collected at Day 4. (A) The number of total BMN cells of femurs and tibias were counted on hemacytometer. The cell number in MP or LSK population was determined by FACS analysis. Data are presented as mean ± S.D. (n = 3) (*p < 0.05) (B) Representative FACS plots for Sca-1 vs c-Kit in lineage-negative population of BMN cells are shown. The gates for MP and LSK and percentages of each population are indicated.
(TIF)

Figure S4 **Either** ***Tab1*** **or** ***Tab2*** **is not essential for HSC function.** (A) 2×10^5^ BMN cells from *Tab1^FF^*, *Tab1^iKO^*, *Tab2^FF^*, or *Tab2^iKO^* mice (CD45.2^+^) were transplanted into lethally irradiated recipients (CD45.1^+^) together with 2×10^5^ competitor wild type BMN cells (CD45.1^+^). At six weeks post transplantation, the chimerism of myeloid, T and B cells in the recipients’ PB was analyzed, then the recipients were i.p. injected with tamoxifen (160 mg/kg body weight) for three consecutive days. The chimerism of PB cells was monitored every three weeks. In each experiment, a CD45.1/CD45.2 mixture of BMN cells was transplanted into two recipients, and the average of their blood cell chimerism is shown. (B) Splenocytes from control No-Cre or *Tab2^iKO^* transplanted mice (#1 and #2) at 21 weeks post-tamoxifen injection were sorted into the CD45.1^+^ or CD45.2^+^ population. Total cell lysates from the sorted splenocytes were analyzed by SDS-PAGE and Western blotted with indicated antibodies. The positions of molecular weight markers are shown on the right. The asterisks indicate the non-specific bands.
(TIF)

Figure S5 **Deletion of** ***Tak1*** **or** ***Tab1/Tab2*** **does not impact the number of B and T cell and granulocyte.** Mice with the indicated genotype (the same animals used for the *in vitro* LSK expansion assay in Figure 3) were i.p. injected with tamoxifen (160 mg/kg body weight) for three consecutive days, and BMN cells were collected at Day 4. BMN cells were analyzed by FACS to determine the number of B cell (B220^+^), T cell (CD3ε^+^) and granulocyte (CD11b^+^ and Gr-1^+^) per femurs and tibias. Data are presented as mean ± S.D (n = 3)
(TIF)

Figure S6 **Cell sorting strategy and efficiency.** Presort gates were set around splenocytes, then CD45.1+CD45.2- and CD45.1-CD45.2+ cells were sorted. The sorting yielded 93 to 99% purity of target populations. Whole cell extracts were prepared from the sorted cells and subjected to Western blot analysis shown in Figure 5C.
(TIF)
