# Supplementary figures and images for: TAK1 (MAP3K7) Signaling Regulates Hematopoietic Stem Cells through TNF-Dependent and -Independent Mechanisms
Source: PLoS One. 2012 Nov 30;7(11):e51073. doi: 10.1371/journal.pone.0051073 (PMC3511369; doi:10.1371/journal.pone.0051073)

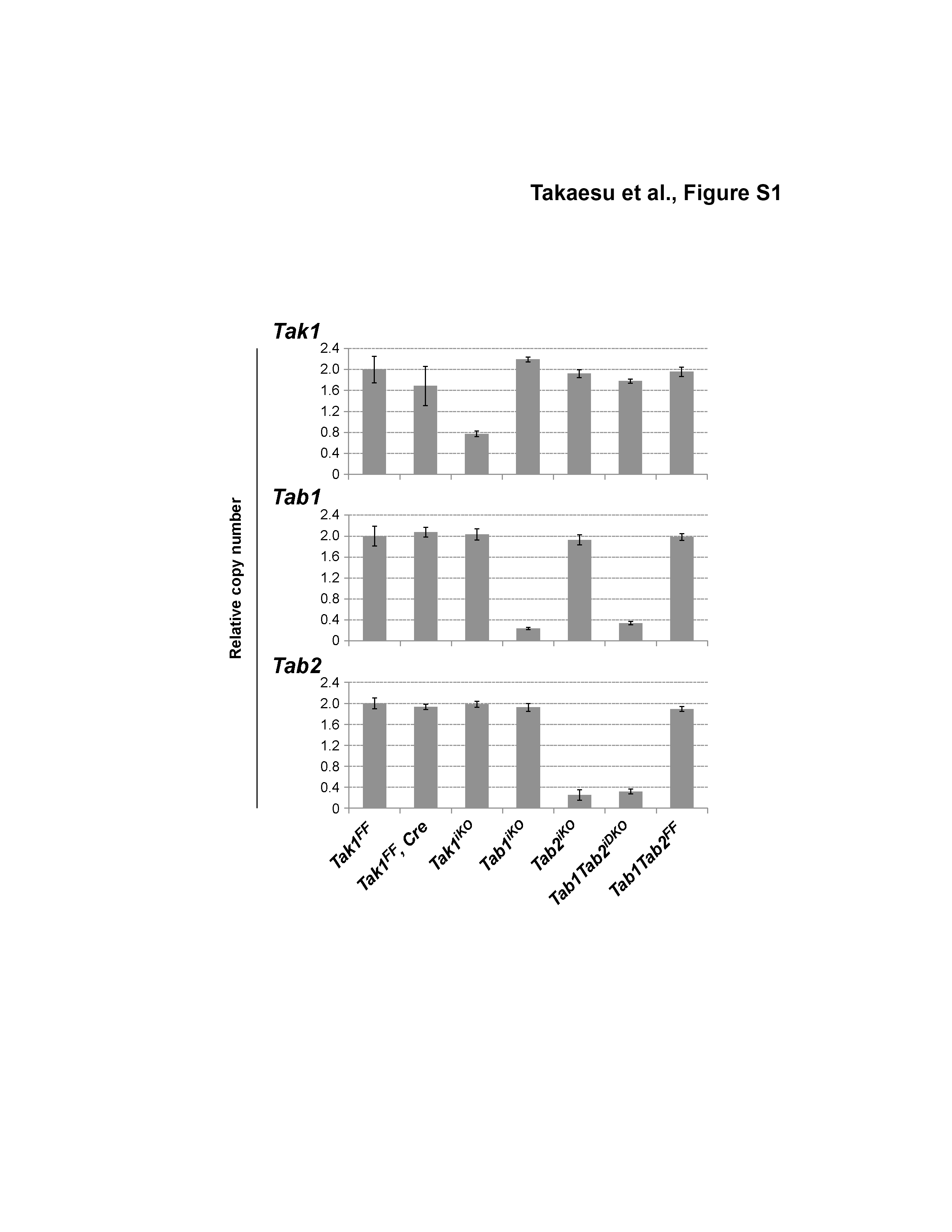

Supplement: Figure S1 — Gene deletions of Tak1 , Tab1 and Tab2 in BMN cells. Mice were i.p. injected with tamoxifen at 160 mg/kg body for three consecutive days (Tak1iKO, Tab1iKO, Tab2iKO and Tab1Tab2iDKO) or untreated (Tak1FF, Tak1FF Cre or Tab1Tab2FF) and sacrificed on day 4. Genomic DNA isolated from BMN cells was analyzed by qPCR using primers designed to detect a portion of the genome flanked by two loxP sites to determine the relative copy number of intact Tak1, Tab1 or Tab2 genome. Data are presented as mean ± S.D. of three independent experiments. (TIF) [file pone.0051073.s001.tif]

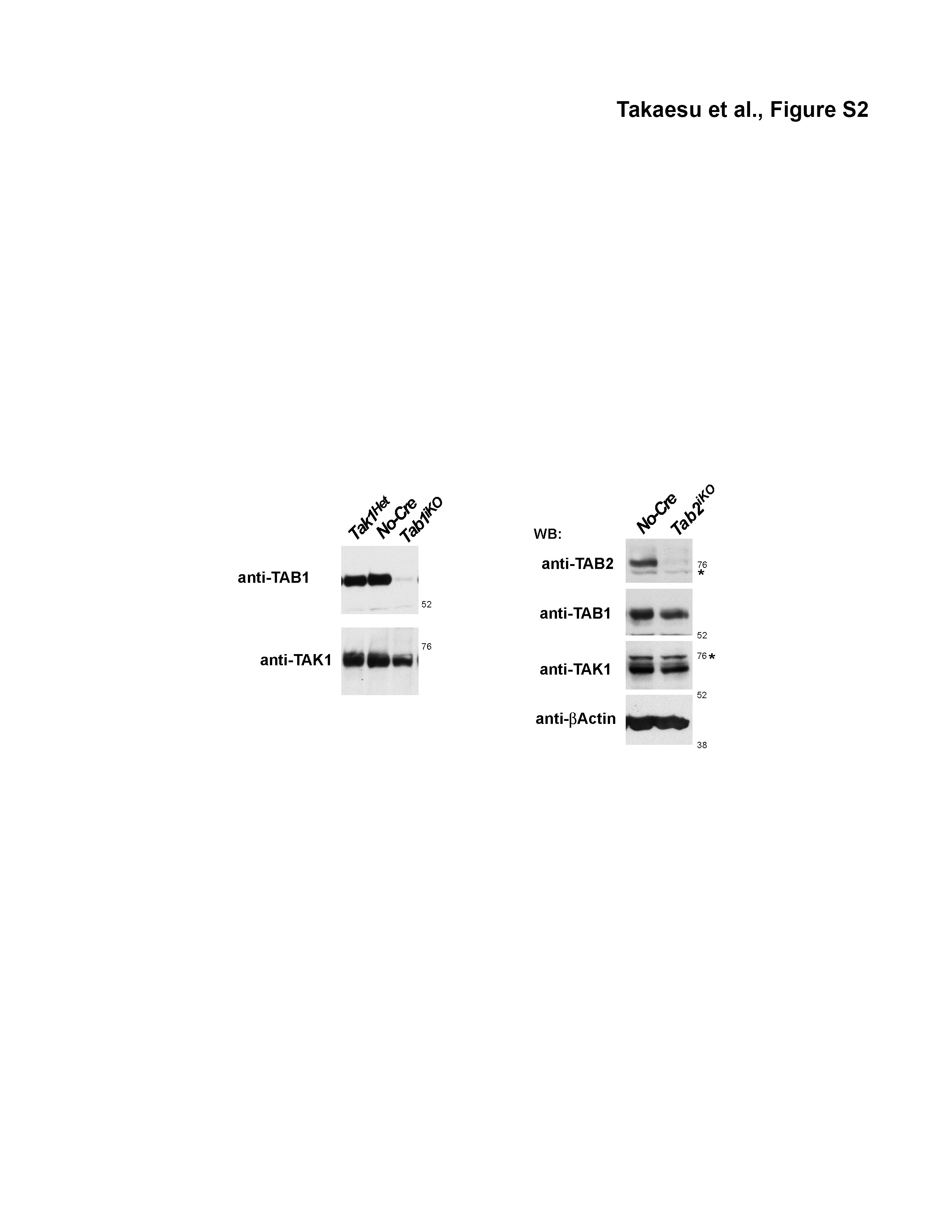

Supplement: Figure S2 — Deletion of TAB1 and TAB2 in splenocytes. Mice with the indicated genotype were i.p. injected with tamoxifen (160 mg/kg) for three consecutive days, and splenocytes were collected at Day 14 (left panels) or Day 4 (right panels). Whole cell extracts were prepared and analyzed by Western blot using the indicated antibodies. Asterisks indicate non-specific bands. (TIF) [file pone.0051073.s002.tif]

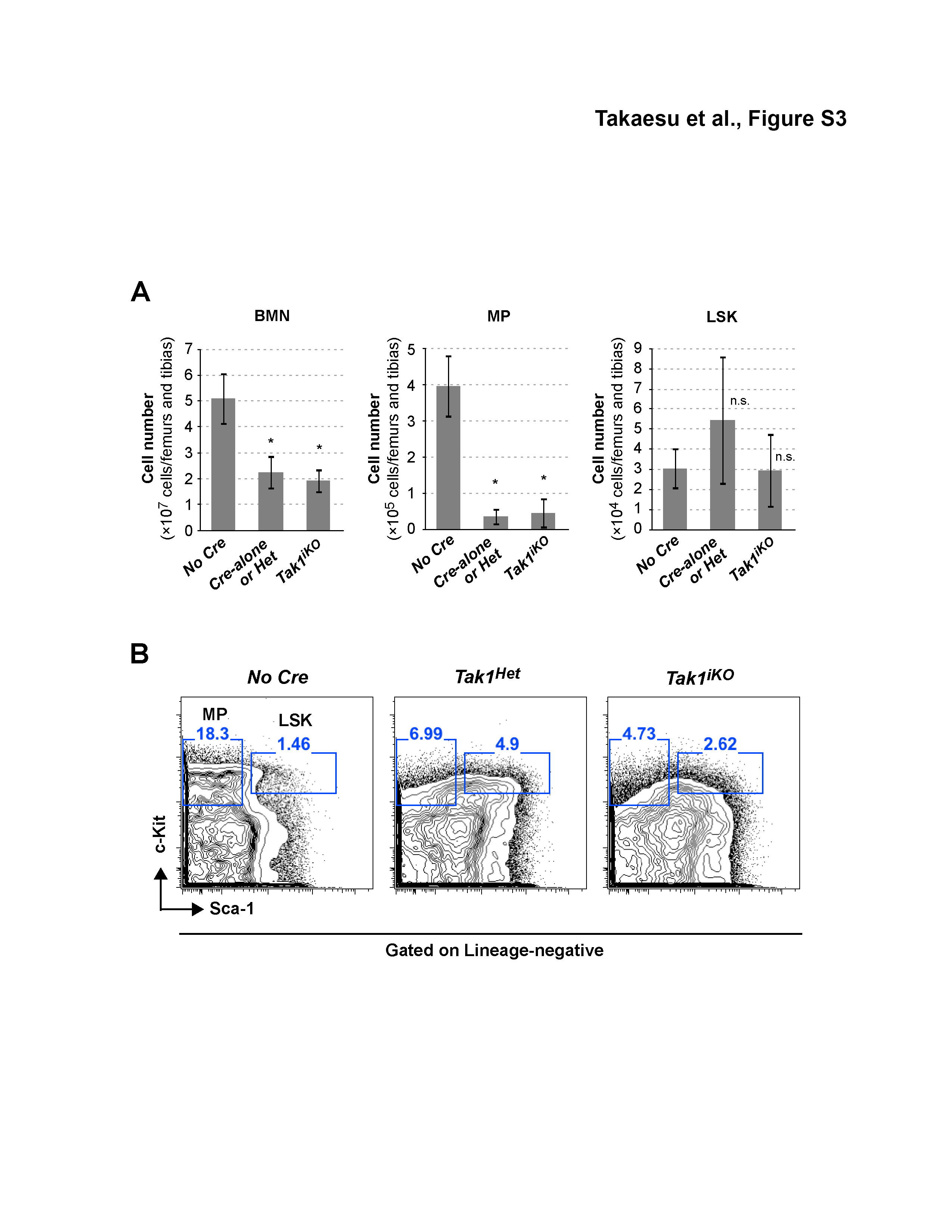

Supplement: Figure S3 — Effect of CreERT activation on hematopoietic cells. Mice with the indicated genotype were i.p. injected with tamoxifen (160 mg/kg body weight) for three consecutive days, and BMN cells were collected at Day 4. (A) The number of total BMN cells of femurs and tibias were counted on hemacytometer. The cell number in MP or LSK population was determined by FACS analysis. Data are presented as mean ± S.D. (n = 3) (*p < 0.05) (B) Representative FACS plots for Sca-1 vs c-Kit in lineage-negative population of BMN cells are shown. The gates for MP and LSK and percentages of each population are indicated. (TIF) [file pone.0051073.s003.tif]

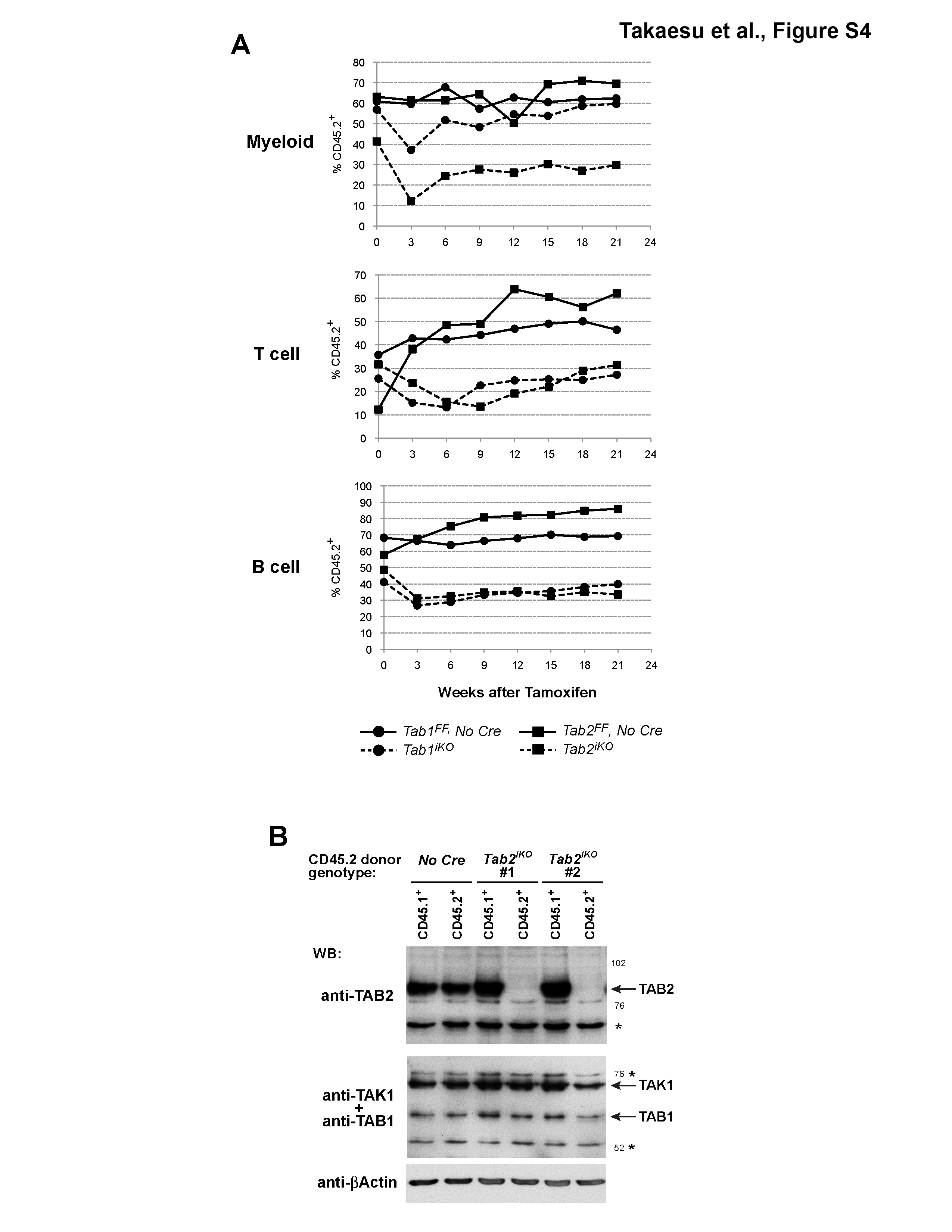

Supplement: Figure S4 — Either Tab1 or Tab2 is not essential for HSC function. (A) 2×10^5^ BMN cells from Tab1FF, Tab1iKO, Tab2FF, or Tab2iKO mice (CD45.2+) were transplanted into lethally irradiated recipients (CD45.1+) together with 2×10^5^ competitor wild type BMN cells (CD45.1+). At six weeks post transplantation, the chimerism of myeloid, T and B cells in the recipients’ PB was analyzed, then the recipients were i.p. injected with tamoxifen (160 mg/kg body weight) for three consecutive days. The chimerism of PB cells was monitored every three weeks. In each experiment, a CD45.1/CD45.2 mixture of BMN cells was transplanted into two recipients, and the average of their blood cell chimerism is shown. (B) Splenocytes from control No-Cre or Tab2iKO transplanted mice (#1 and #2) at 21 weeks post-tamoxifen injection were sorted into the CD45.1+ or CD45.2+ population. Total cell lysates from the sorted splenocytes were analyzed by SDS-PAGE and Western blotted with indicated antibodies. The positions of molecular weight markers are shown on the right. The asterisks indicate the non-specific bands. (TIF) [file pone.0051073.s004.tif]

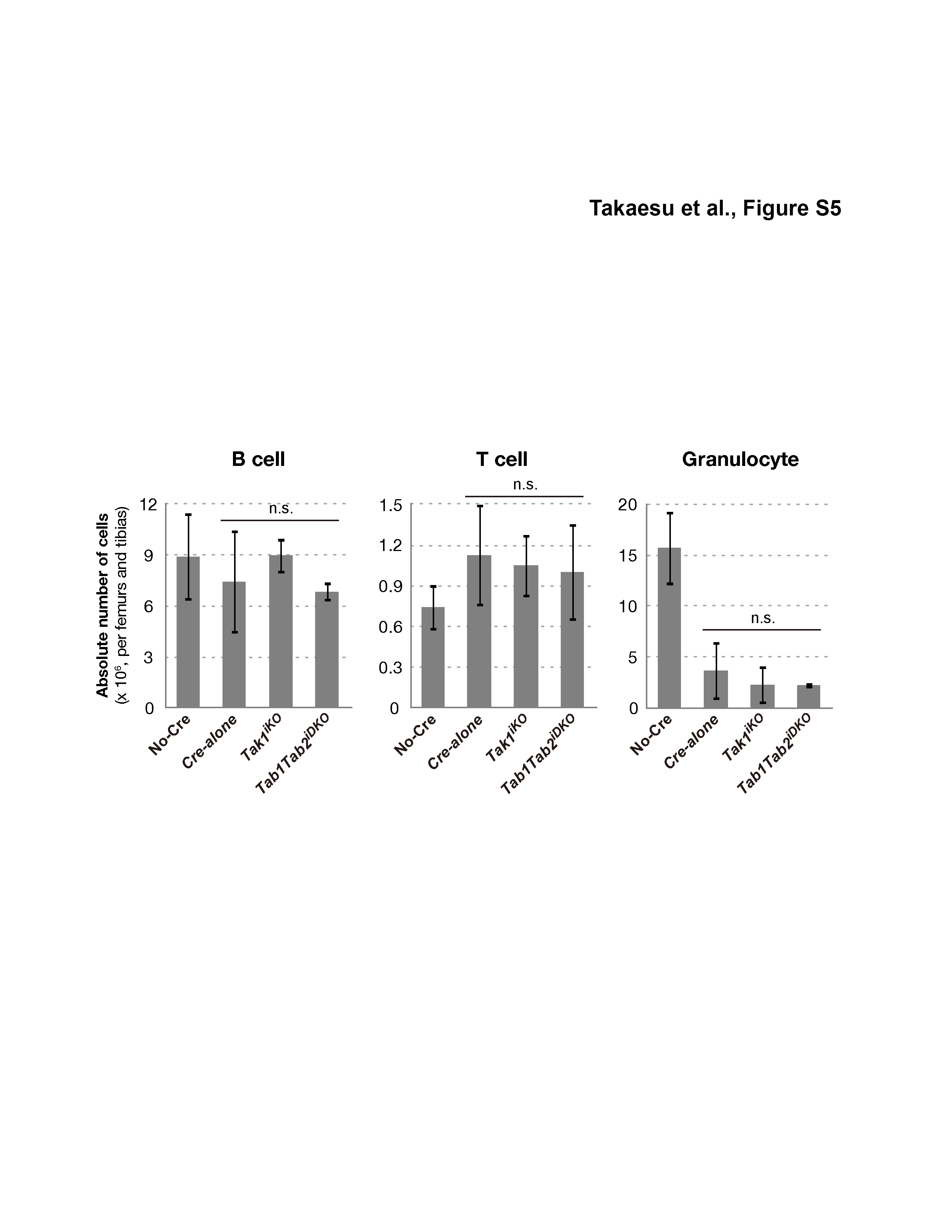

Supplement: Figure S5 — Deletion of Tak1 or Tab1/Tab2 does not impact the number of B and T cell and granulocyte. Mice with the indicated genotype (the same animals used for the in vitro LSK expansion assay in Figure 3) were i.p. injected with tamoxifen (160 mg/kg body weight) for three consecutive days, and BMN cells were collected at Day 4. BMN cells were analyzed by FACS to determine the number of B cell (B220+), T cell (CD3ε+) and granulocyte (CD11b+ and Gr-1+) per femurs and tibias. Data are presented as mean ± S.D (n = 3) (TIF) [file pone.0051073.s005.tif]

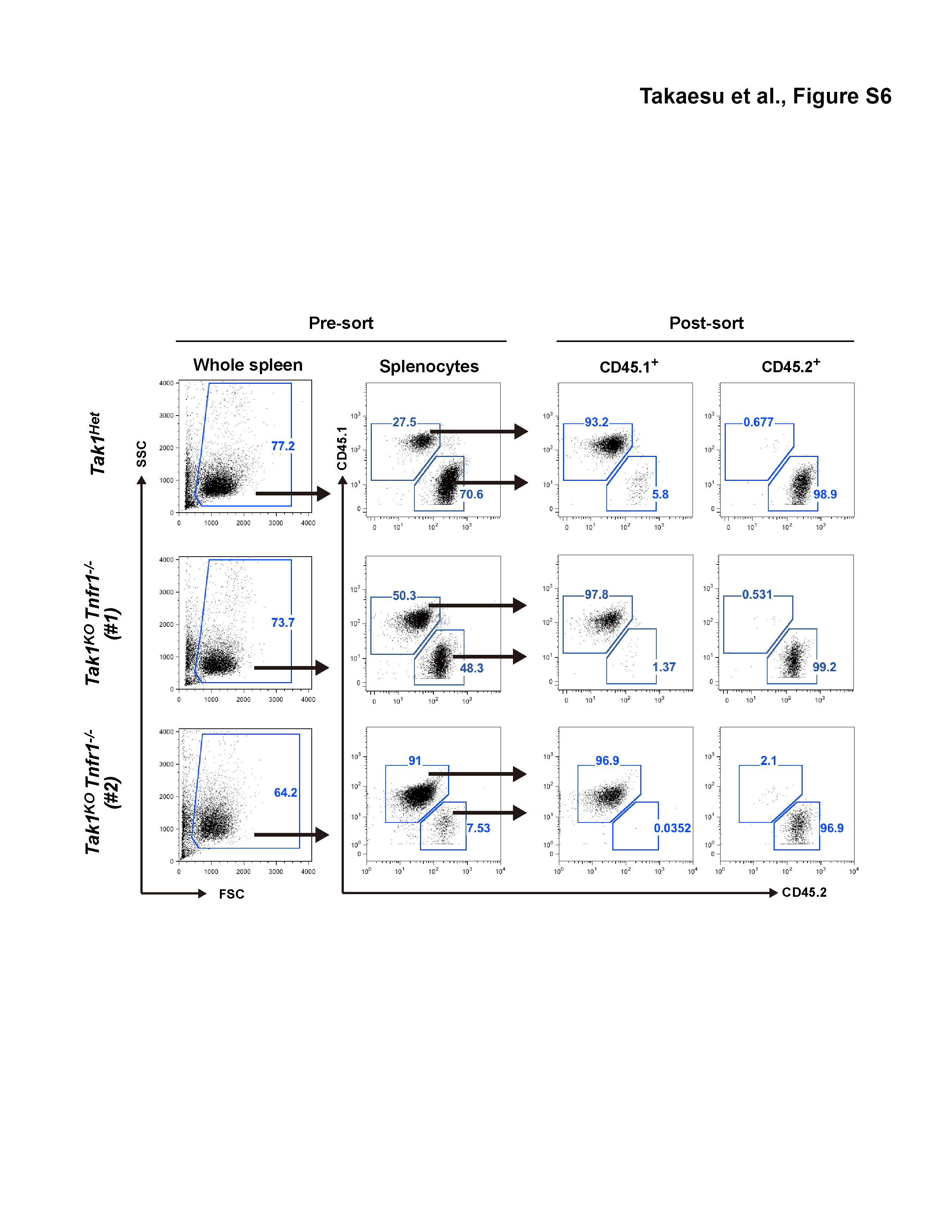

Supplement: Figure S6 — Cell sorting strategy and efficiency. Presort gates were set around splenocytes, then CD45.1+CD45.2- and CD45.1-CD45.2+ cells were sorted. The sorting yielded 93 to 99% purity of target populations. Whole cell extracts were prepared from the sorted cells and subjected to Western blot analysis shown in Figure 5C. (TIF) [file pone.0051073.s006.tif]
